# Tumor-associated macrophages induce vasculogenic mimicry of glioblastoma multiforme through cyclooxygenase-2 activation

**DOI:** 10.18632/oncotarget.6930

**Published:** 2016-01-18

**Authors:** Xiaoming Rong, Bo Huang, Shuwei Qiu, Xiangpen Li, Lei He, Ying Peng

**Affiliations:** ^1^ Department of Neurology, Sun Yat-sen Memorial Hospital, Sun Yat-sen University, Guangzhou 510120, China; ^2^ Department of Neurology, The First Affiliated Hospital of Soochow University, Suzhou 215006, China

**Keywords:** M_2_ macrophages, glioblastoma multiforme, vasculogenic mimicry, COX-2

## Abstract

Glioblastoma multiforme (GBM) is a malignant brain tumor with characteristics of strong aggressiveness which depend on vigorous microvascular supply. Vasculogenic mimicry (VM), a new microvascular circulation not involving endothelial cells, is reported as one part of the vascularization of GBM. Tumor-associated macrophages (TAMs), mostly present as immunosuppressive M_2_ phenotype in GBM, are well known as a promoter for tumor angiogenesis. However, whether TAMs can induce VM in GBM remains uncertain. In the present study, immunohistochemistry showed that higher numbers of macrophages infiltrating in the VM-positive area where tumor cells also highly express COX-2. By using the coculture model of U87 cell line and Interleukin-4-activated M_2_ macrophages, we found that the capability of VM formation was increased and COX-2 expression was up-regulated in U87 cells. Moreover, knockdown of COX-2 by siRNA Oligonucleotides or abrogating activity of COX-2 by specific inhibitors resulted in impairment of VM formation. Besides, in the process of VM formation, PGE_2_/EP_1_/PKC pathway was activated in U87 cells and inhibition of COX-2 led to down-regulation of PGE_2_ and PKC. In *in vivo* experiment, we found that COX-2 loss of function in the U87 xenograft model lead to less vascular mimicry. Collectively, our study demonstrates that M_2_ macrophages are capable of promoting generation of VM in GBM with COX-2 dependent, providing potential mechanisms of the interaction between inflammatory microenvironment and perivascular microenvironment.

## INTRODUCTION

Glioblastoma multiforme (GBM) is the most common malignant primary brain tumor in adults, with characteristics of extreme aggressiveness and high proliferation[1]. Irrespective of surgical resection and radiotherapy/chemotherapy, its median survival remains to be about 14.6 months[2]. The aggressiveness of GBM depends not only on the invasion of tumor cells and extracellular matrix remodeling, but also on the strong vascular and nutrition supply to the tumor cells. However, anti-angiogenic monotherapy, for example bevacizumab (a monoclonal antibody of vascular endothelial growth factor), did not show anticipated benefit for improving the overall survival[3, 4]. Some patients still had tumor recurrence. For those suffered from GBM recurrence, bevacizumab revealed little therapeutic effect[5, 6]. The above results remind us that there may be another vascular supply system which is different from the classic blood vessels dependent on vascular endothelial cells.

Vasculogenic mimicry (VM), first described in human melanoma, is a new blood supply system that tumor cells generate vascular-like channels to facilitate tumor perfusion independent of endothelial cell angiogenesis[7]. In these patterned channels, red cells are detected within the channels while endothelial cell markers such as CD31, CD34, factor VIII-related antigen are not identified. Recently, a number of research have demonstrated that the proportion of VM to the overall vascular channels in GBM is 23% - 55%[8, 9]. The malignancy of GBM is reported to be positively correlated with the proportion of VM[8, 10]. Growing evidence also suggest that VM is ascribed to the capability of tumor cells to transdifferentiate into non-endothelial cells.

Tumor-associated macrophages (TAMs), the dominant tumor-infiltrating inflammatory cells, have been linked to the promotion of tumor growth through inducing angiogenesis, invasion and matrix remodeling. The majority of TAMs locate around the glioma stem cells (GSCs) and play an important role on the maintenance of GSCs self-renewal and plasticity[11]. Since inflammatory microenvironment is involved in angiogenesis and plasticity of tumor cells, we presumed that inflammatory microenvironment might also promote VM formation. Previous studies reported that cocultured with TAMs, the expression of cyclooxygenase-2 (COX-2) in basal cell carcinoma cells would significantly increase[12]. COX-2 can catalyze the conversion of arachadonic acid into prostaglandin H_2_, which subsequently convert into primary prostaglandin E_2_ (PGE_2_). Both COX-2 and PGE_2_ are highly expressed in extremely aggressive tumors and are associated with invasion and angiogenesis[13, 14]. Interestingly, in breast cancer, tumor cells that over-express COX-2 have the unique ability to form VM and their activity of signaling pathway COX-2/PGE_2_/EP_3_ are activated[15]. However, whether TAMs can directly induce VM formation in GBMs is still unknown. In this study, we evaluated the relationship between macrophages infiltration and VM expression in glioma samples and then took advantage of coculture model of U87 cells and Interleukin-4 (IL-4)-activated M_2_ macrophages to investigate whether TAMs could enhance the ability of GBM cells to generate VM. In addition, we sought to identify the candidate pathway that involved in TAM-induced VM formation.

## RESULTS

### Higher numbers of macrophages infiltrate in VM-positive area in GBM

**Figure 1:**
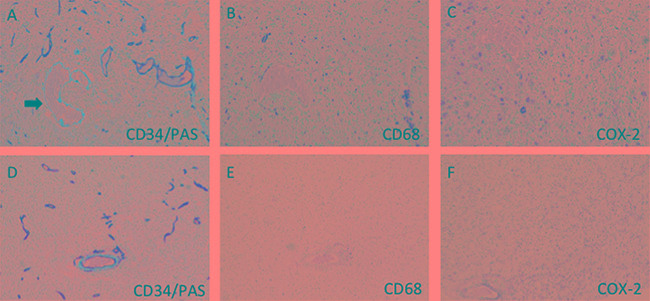
Immunohistochemistry of VM and macrophages in GBM samples **A.** Representative VM (arrow) and vessels (brown staining) in GBM. The VM was CD34^−^/PAS^+^ and showed purple, while the vessels were CD34^+^/PAS^+^and showed brown and purple. VM-positive regions showed much more numbers of macrophages **(B)** than that in VM-negative areas **(E).** In addition, VM-positive area revealed much higher COX-2 expression than those in VM-negative area **(C, F).** Original magnification: x200.

**Figure 2:**
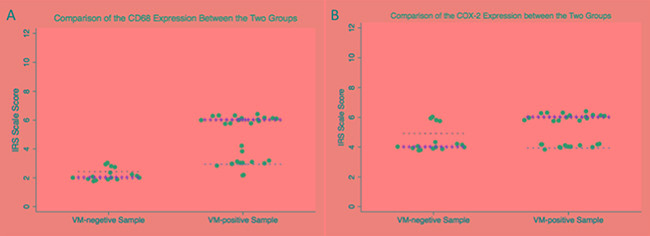
Comparison of IRS score in CD68 and COX-2 between VM-positive samples and VM-negative samples

To evaluate the presence of VM structures in GBM, we examined tumor samples from forty-four GBM cases. Vascular basement membrane was stained by PAS, and most of these vessels exhibited strong staining of CD34 (a vascular endothelial cell marker, Figure 1A). In contrast to the vessels that consisted of endothelial cells, VM were positive for PAS but negative for CD34 (Figure 1A). Of all the 44 GBM samples, 29 were found to have VM. To determine whether VM structure was correlated with macrophages infiltration, we stained the sample with CD68, a marker of macrophages. Immunohistochemistry (IHC) analysis revealed that more macrophages infiltrating in VM-positive areas than that in VM-negative areas (Figure 1B, 1E, Figure 2A). In addition, increased expression of COX-2 was found in the tumor cells near VM positive area (Figure 1C, 1F, Figure 2B). These results indicated that VM tend to present in GBM area where tumor cells highly express COX-2 and a relatively large number of macrophages infiltrated.

### IL-4 activates macrophages to a TAM-like phenotype

**Figure 3:**
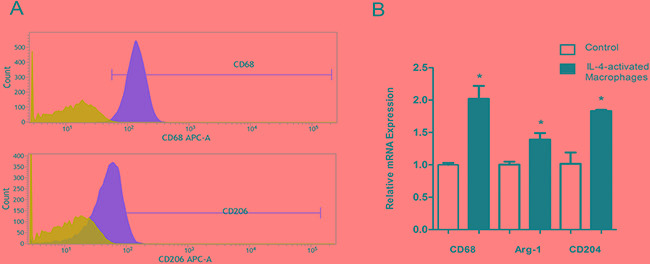
IL-4 activates macrophages to a M_2_ Phenotype **A.** Monocyte/macrophages treated with 40ng/ml IL-4 for 72 hours showed significant induction for CD 68 (marker for macrophages), CD 206 (marker for M_2_ macrophages). **B.** qPCR showed that the mRNA expression of Arg-1 and CD204 (both markers for M_2_ macrophages) were also increased compared to the control group.

In order to further study the role of macrophages on VM formation, we use the *in vitro* coculture model. Since most of macrophages in the tumor microenvironment were induced into immunosuppressive M_2_ phenotype, we firstly isolated monocytes from peripheral blood mononuclear cells (PBMC), and continue to culture them in DMEM with 10% fetal bovine serum (FBS). Monocytes became attached and then differentiated into macrophages. Three days later, medium was changed and 40ng/ml IL-4 was added into the medium for further 72 hours. Macrophages treated with IL-4 became stretched and elongated and exhibited a CD68 high and CD206 high phenotype (Figure 3A), similar to the previous studies[16]. Consistent with the changes in flow cytomatry, m-RNA level of CD68, Arg-1, CD204 also significantly increased in IL-4-treated macrophages (Figure 3B).

### IL-4-activated macrophages induce vasculogenic mimicry in GBM cells

**Figure 4:**
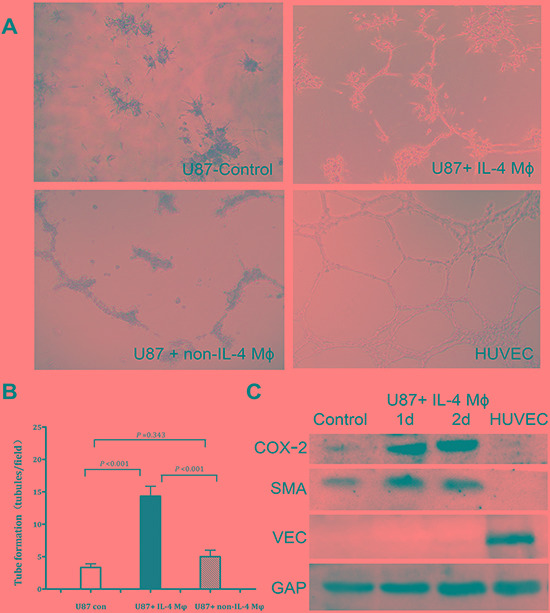
IL-4-activated macrophages induce vascular tube formation in U87 **A.** U87 cells were plated on matrigel for tube formation. HUVEC served as a positive control. **B.** Tubules formed by U87 cells were quantified. Total number of tubules in x100 view was comparedamong the three groups. The results revealed that U87 cocultured with M_2_ macrophages were much easier to form vascular-like channels compared with the other two groups. **C.** Western blot showed that coculturing with M_2_ macrophages could increase COX-2 and SMA expression in U87 cells, while VE-cadherin expression was bare to detect.

To test our hypothesis that IL-4-activated macrophages could enhance the ability of GBM cells in developing vascular-like channels, we took advantage of coculture model of U87 cells and IL-4-activated M_2_ macrophages and then performed a tube formation assay that recapitulated the ability of vascular endothelial cells to develop vasculature *in vitro*. We found that U87 cells, after coculturing with M_2_ macrophages, developed much more network as compared with those without coculture (Figure 4A, 4B). Moreover, U87 cells which cocultured with non-IL-4 macrophages also formed a capillary phenotype in matrigel, although the ability was relatively weaker. We further tested the CD68 and CD206 expression in those non-IL-4-activated macrophages in coculture model. Flow cytometry showed that these macrophages also highly expressed CD68 and CD206, which supported the previous founding that tumor cells has the ability to drive macrophages to M_2_ phenotype.[17, 18] To further determine whether these cocultured tumor cells acted as mural-like cells, we tested the protein expression of smooth muscle α-actin (SMA, specific marker of mural cells) and VE-cadherin (specific marker of vascular endothelial cells) in our target cells. We found that U87 cells cocultured with M_2_ macrophages expressed stronger SMA than those without coculture (Figure 4C), while the VE-Cadherin expression was quite low. In contrast, HUVEC showed high level of VE-Cadherin and barely detectable level of SMA. These results supported our hypothesis that M_2_ macrophages promoted vasculogenic mimicry formation in GBM cells.

### Induction of VM formation in GBM cells by M_2_ macrophages is COX-2 dependent

**Figure 5:**
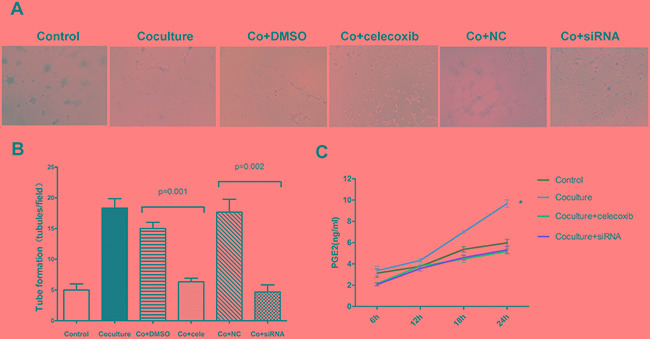
The promotion of VM in U87 cells by M_2_ macrophages depends on COX-2 high expression **A.** U87 cells with or without coculture with M_2_ macrophages were used for tube formation in the presence of celecoxib (20nM) and siRNA technology. **B.** Tubules were quantified. Total number of tubules in x100 view was compared among the groups. Compared with DMSO treatment, celecoxib could significantly inhibit the VM formation in U87 cells cocultured with M_2_ macrophages (6.3 ± 0.6 *vs.* 15.0 ± 1.0, *p* = 0.001). Similarly, treatment with siRNA also suppressed the ability to form channels in U87 cells as compared with NC treatment (4.7 ± 1.2 *vs.* 17.7 ± 2.1, *p* = 0.002). **C.** PGE_2_ expression was increased after being cocultured with M_2_ macrophages and the course could be abrogated by celecoxib and siRNA the. (*, the technology change of PGE_2_ concentration at 24h from 6h in coculture group was much larger then that in control group, *p* = 0.014). Abbreviation: co=coculture, cele=celecoxib.

Besides SMA, western bolt revealed that COX-2 expression was also up-regulated in U87 cells after coculture (Figure 4C). In order to confirm the role of COX-2 in VM formation, we employed a COX-2 specific inhibitor celecoxib. Considering celecoxib decreases cell proliferation as well as COX-2 expression at a dose-dependent manner, we firstly tested both proliferation ability and COX-2 protein level at a dose range from 10nM to 40nM. Our results revealed that a dose of larger than 30nM could significantly destroy the proliferation ability of U87 cells. The optimal dose of 20nM celecoxib was based on mild destroy of proliferation accompanied with effective decrease of COX-2 level in U87 cells, and this dose was used in the subsequent experiments. VM assay showed that celecoxib treatment was able to significantly reduce the number of vascular-like channel formed by U87 cells which were cocultured with M_2_ macrophages as compared with those treated with vehicle (6.3 ± 0.6 *vs.* 15.0 ± 1.0, *p* = 0.001, Figure 5A–5B). To verify our observation with celecoxib, we silenced the expression of COX-2 in U87 cells by siRNA technology. Suppression of COX-2, the tubules formed by U87 cells were decreased by about 74% (4.7 ± 1.2 *vs.* 17.7 ± 2.1, *p* = 0.002, Figure 5A–5B). The above findings suggested that M_2_ macrophage could induce VM formation in U87 cells and this event was correlated with high expression of COX-2.

### M_2_ macrophages activate COX-2/PGE_2_/EP_1_/PKC pathway of U87 cells

**Figure 6:**
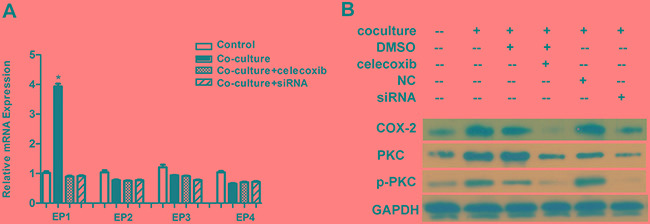
M_2_ macrophages activate a COX-2/EP_1_/PKC cascade in U87 cells **A.** The EP_1_ mRNA level in U87 cells was largely increased after being cocultured with M_2_ macrophages (*, compared with control group, *p* = 0.001), while other EP receptors (EP_2-4_) did not show significant changes. **B.** Western blot showed that PKC and *p*-PKC were up-regulated in U87 cells after being cocultured with M_2_ macrophages, and these changes were also dependent on high COX-2 expression.

Previous study reported that COX-2 could activate protein kinase C (PKC) and lead to a series of downstream reaction[19]. Here we determined whether COX-2/PGE_2_/EP/PKC pathway was activated in U87 cells after being cocultured with M_2_ macrophages. Firstly, elisa assay confirmed the increase of PGE_2_ in U87 cells in coculture model (Figure 5C). Different from previous studies, EP_1_ receptor but not EP_3_ or EP_4,_ was found to be significantly up-regulated after coculture by using q-PCR analysis (Figure 6A). Moreover, the PKC and *p*-PKC protein expression in U87 cells also markedly increased (Figure 6B). Treatment with celecoxib or COX-2 siRNA could suppress the up-regulation of PGE_2_ as well as the activation of PKC.

### Celecoxib inhibits VM formation in xenograft model

**Figure 7:**
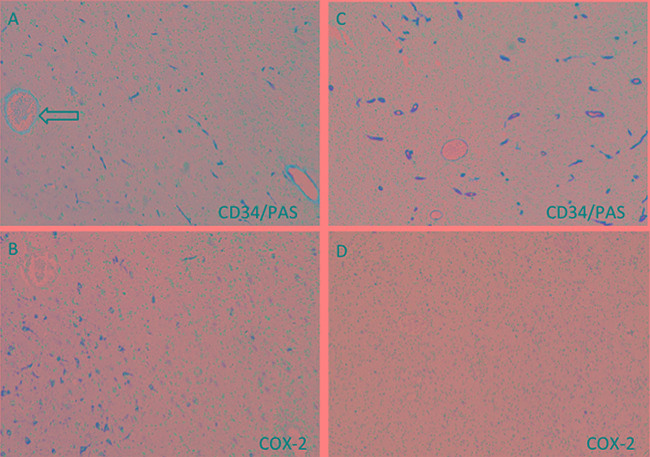
Suppression function of COX-2 in the U87 xenograft model does indeed lead to less vascular mimicry Thedensity of PAS+/CD34- structures in control tumors were about 2.9-fold greater than those found in celecoxib-treated tumors (11.0% vs. 3.8%, *p* = 0.0124). In VM positive area (**A**, arrow), the COX-2 staining reaction was much stronger **(B)** than those in VM-negative area **(C, D).**

In an attempt to establish a functional role for COX-2 in the development of VM, we utilized a tumor xenograft model by inplanting U87 cells into BABIC mice. Mice were divided into two groups: U87 wild type cells without treatment and U87 wild type cells treated with celecoxib. Celecoxib was begin seven days after transplantation and lasted for concurrently 2 weeks. All mice were sacrificed at the end of the third weeks and brain was removed for analyzing tumor vasculogenesis. IHC analysis revealed that both the control tumor and celecoxib-treated tumor harbored CD34-positive vascular channels and CD34-negative VM structures. However, the density of PAS+/CD34- structures in control tumors were about 2.9-fold greater than those found in celecoxib tumors (Figure 7A, 7C). We further revealed a high expression of COX-2 near VM structure, which was similar as the results in human GBM samples (Figure 7B, 7D). Collectively, the *in vivo* data indicate that the formation of VM in GBM depends on COX-2 high expression.

### COX-2 expression is correlated with progression-free survival of GBM patients

**Figure 8:**
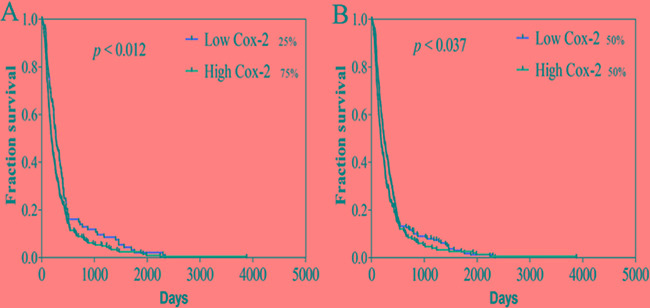
Survival curves of GBM patient related with COX-2 expression Progression-free survival (PFS) was analyzed using the Kaplan-Meier method at 25% **A.** and 50% **B.** quartiles of Cox-2 expression. Low COX-2 was related with long PFS among the 458 GBM patients at the indicated cut-off *p* < 0.037). The log-rank test *P* value for the difference between two survival curves for low and high COX-2 expression in GBM patients was indicated.

Given the importance of COX-2 in VM formation, survival analysis related with COX-2 was performed by using TCGA dataset including 458 GBM patients. We found that low expression of COX-2 was significantly correlated with extended progression-free survival (PFS) of GBM patients at different stratifications (Figure 8; 25% quartile *vs.* 75% quartile: 381.7 ± 443.2 *vs.* 286.1 ± 365.7 days; 50% quartile: 342.3 ± 384.3 *vs.* 277.2 ± 390.1 days) after adjusting for age and gender. Although indirectly, these data suggested that VM formation and COX-2 expression is involved in progression of GBM.

## DISCUSSION

Our study showed the infiltration of macrophages in VM-positive areas and the role and underlying mechanism of M_2_ macrophages in inducing VM formation in GBM cells. Since the first introduction in 1999, VM has been proved in a variety of malignant tumors, including aggressive breast cancer, ovarian cancer and high malignant glioma [15, 20]. In Wang's study, immunohistochemical assay was taken in 86 patients with primary astrocytoma, and 23 of them were VM positive[10]. The glioblastoma tissues were more likely to have lower microvessel density and necrosis in the VM-rich area, which indicates that VM is an effective nutrition supply independent on normal vasculature. Our study showed a 65.9% VM positive rate in GBM, which is consistent with the previous studies[8, 10]. Growing evidence found that not all the glioma cells can generate VM[21–23]. Highly aggressive tumor cells line U251 and U87 were reported to be able to form VM, while lower malignant cells line, such as SHG44, could not generate VM even after adding conditional medium from highly invasive and metastatic cell lines[7]. Except for the grade of tumor, the local vascular system is regulated by the surrounding microenvironment. Inflammation is well known as an inducer for angiogenesis. Piao *et al* demonstrated that a majority of macrophages can be recruited by local hypoxia due to antiangiogenic agents[24]. By producing a number of cytokins, macrophages have been shown to maintain GSCs and promote tumor progression. However, no relationship was demonstrated between macrophages and VM. Through immunohistochemistry and CD34/PAS double-staining, we found that more macrophages infiltrated in VM-positive areas. In order to investigate whether M_2_ macrophages promote VM formation in GBM cells, we compared the capability of channel formation in U87 cells with or without coculture with M_2_ macrophages. The results revealed that M_2_ macrophages could enhance the ability to generate vascular channels in U87 cells within normal oxygen. The high expression of SMA and barely detectable of VE-cadherin in U87 cells in our model proved that these vascular channels consisted of mural-like cells transdifferenciated from tumor cells. Apart from mural-like cells, GBM cells can also transdifferentiate into vascular endothelial cells. However, this procedure is usually driven by hypoxia.[25–27]. Different from VM, tumor-derived endothelial cells show the same gene expression with regular endothelial cells, e. g. CD31, vWF, VE- cadherin. Our results supported that in normal oxygen microenvironment, GBM cells tend to trandifferentiate into mural like cells that participate in angiogenesis.

Previous studies found that proinflammatory genes such as COX-2, NF-kB in TAMs infiltrating regions are significantly enhanced[28].To gain additional insight into the mechanism by which M_2_ macrophages promote VM formation in U87 cells, we assessed the proinflammatory genes COX-2 expression in the coculture model. The results shown that COX-2 expression and its downstream PGE_2_ secretion were up-regulated in U87 cells after being cocultured with M_2_ macrophages. Further more, by using COX-2 selective inhibitor celecoxib and RNA interference, the capability of VM formation was markedly suppressed, which indicated that the promotion of VM in U87 cells by M2 macrophages is depend on upregulation of COX-2 activation. The positive correlation of COX-2 expression and VM percentage in animal model and the predictive effect of high COX-2 expression in shorten progression-free survival in GBM patients also supported the role of COX-2 in stimulating tumor progression. Previously, a growing evidence suggests that COX-2 is involved in tumor proliferation, invasion and angiogenesis[14, 29]. In invasive breast cancer, knockdown of COX-2 inhibited VM which could be rescued with PGE_2_[15, 30]. Prostanoid receptor 3 (EP_3_) was the major receptor to regulate VM network in breast cancer. However, in our study, only EP_1_ expression was found to significantly increased after coculture. We assumed that the activated EP receptors might not be the same among different tumors in VM formation. Our results also found that the PKC pathway in U87 cells was activated in the coculture model. By suppressing the expression and activation of COX-2, PGE_2_/PKC level was also reduced accompany with fewer vascular-like channel formation. We presumed that activated PKC might promote VM through promoting VEGF and VM-associated gene expression, as well as by activating extracellular signal-regulated kinase.

In sum, our study found that M_2_ macrophages could induce VM via up-regulating COX-2 expression in GBM cells. It indicates a new crosstalk between inflammatory microenvironment and perivascular microenvironment, and suggests that the promotion of VM by macrophages may limit the effectiveness of antiangiogenic agents.

## MATERIALS AND METHODS

### Glioma specimens

We performed a retrospective study of patients who were diagnosed as GBM between January 2013 and December 2014. A total of 44 paraffin-embedded GBM tissues were obtained from the Department of Pathology of Sun Yat-sen Memorial Hospital. The tumor sections were reviewed by pathologists to verify the diagnosis of GBM according to the 2007 World Health Organization classification of central nervous system tumors. The study was approved by the ethics committee at Sun Yat-sen Memorial Hospital and all patients gave informed consent.

### Cell culture

The human U87 cells line and human umbilical vein endothelial cells line (HUVECs) were obtained from American Type Culture Collection (ATCC). U87 cells were grown in high glucose Dulbecco's Modified Eagle Medium (DMEM) (Gibco, Invitrogen) supplemented with 10% FBS (Gibco, Invitrogen) and 1% penicillin/streptomycin (Gibco, Invitrogen). HUVECs were maintained in RPMI 1640 (Gibco, Invitrogen) supplemented with 10% FBS and 1% penicillin/streptomycin. All the cells were grew in 5% CO_2_ at 37°C.

Fresh human peripheral blood was obtained from Sun Yat-sen Memorial Hospital, according to the guidelines approved by the ethics committee at Sun Yat-sen Memorial Hospital. Monocyte-derived M_2_ macrophages were generated from human PBMC as described previously[12]. Briefly, PBMC were isolated from peripheral blood by density gradient centrifugation. After being purified by using anti-CD14 microbeads (Miltenyi Biotec), monocytes were incubated in DMEM supplemented with 10% FBS and 1% penicillin/streptomycin for 3 days. M_2_ macrophage polarization were obtained by removing the median and culturing cells for another 3 days in DMEM supplemented with 40 ng/ml Recombinant Human IL-4 (PeproTech).

### Macrophages and U87 cells coculture

Twelve-well transwell plates (Corning, LifeScience) were used in coculture model. IL-4-activated M_2_ macrophages (2×10^5^) were seeded into the upper inserts and U87 cells (2×10^5^) were placed in the lower inserts. After 48 hours, the macrophages were discarded, and the U87 cells were washed and used for subsequent experiments.

### Flow cytometry

Monocytes/macrophages with or without treated with IL-4 were washed and resuspended in PBS. For intracellular CD68 staining, cells were fixed and permeabilized with permeabilization reagents (CALTAG^™^ Laboratories), and then incubated with the anti-human CD68-APC (BD Bioscience). For detection of CD206, a surface marker, cells were incubated with CD206-APC (BD Bioscience). After the final washing step, cells were analyzed by flow cytometry (BD FACSVerse Z6511550198).

### Tube formation for vasculogenic mimicry

Tube formation was performed as described previously[22]. Briefly, a 96-well tissue culture plate was evenly coated with 60ul/well matrigel (BD Bioscience) and solidified at 37°C for 30min. U87 cells (2×10^4^) with or without coculturing with M_2_ macrophages were plated on matrigel and HUVECs were used as control. After 24 hours, images of the cells were taken by using inverted fluorescence microscope (Olympus IX71).

### Small interfering RNA transfections

COX-2 siRNA and negative control (NC) were purchased from Shanghai GenePharma Co., Ltd. U87 cells were transfected using the transfection reagent Lipofectamine 2000 (Invitrogen) for 6 hours; medium were then changed and cells continued to be incubated for 24 hours before subsequent experiments.

### RNA extraction and quantitative real-time PCR

Total mRNA and quantitative real-time PCR (q-PCR) was performed according to the classic protocol and the primers of CD68, Arg-1, CD204, EP_1_, EP_2_, EP_3_, EP_4_ were listed in Table 1.

**Table 1:** qPCR primers sequences

CD68	Forward: 5′-GGAAATGCCACGGTTCATCCA-3′
	Reverse: 5′-TGGGGTTCAGTACAGAGATGC-3′
Arg-1	Forward: 5′-GTGGAAACTTGCATGGACAAC-3′
	Reverse: 5′-AATCCTGGCACATCGGGAATC-3′
CD204	Forward: 5′-CCAGGGACATGGGAATGCAA-3′
	Reverse: 5′-CCACTGGGACCTCGATCTCC-3′
EP1	Forward: 5′-AGCTTGTCGGTATCATGGTGG-3′
	Reverse: 5′-AAGAGGCGAAGCAGTTGGC-3′
EP2	Forward: 5′-CGATGCTCATGCTCTTCGC-3′
	Reverse: 5′-GGGAGACTGCATAGATGACAGG-3′
EP3	Forward: 5′-GTCGTCATCGTCGTGTACCTG-3′
	Reverse: 5′-AGTCATGGTCAGCCCGAAAAA-3′
EP4	Forward: 5′-CCGGCGGTGATGTTCATCTT-3′
	Reverse: 5′-CCCACATACCAGCGTGTAGAA-3′

### Western blotting

Total proteins were extracted using RIPA lysis buffer (Pierce). Protein lysates were separated by 8% or 10% SDS-PAGE and then electrophoretically transferred to polyvinyl difluoride membrane. After being blocked in 5% non-fat milk, the membrane was incubated with either VE-Cadherin (1:1000, Cell Signaling Technology), SMA (1ug/ml, Abcam), COX-2 (1:500, Bioworld Technology), PKC-α (1:500, Bioworld Technology), or phospho-PKC-α (1:500, Bioworld Technology). GAPDH antibody (1:2000, Cell Signaling Technology) was used as control.

### Immunohistochemistry and CD34/PAS double-staining

Immunohistochemical and CD34/PAS double-staining were performed according to the conventional protocol. Macrophages were marked by CD68 (Abcam). For CD34/PAS double-staining, after marking for CD34 (Zhongshan Goldenbridge Biotechnology, China), the slides were washed and were stained by PAS. For each tumor sample, we examined 5 randomized areas (200× on a microscope) containing perfused structure and calculated the total number of vessels which were CD34 positive as well as the number of VM structures. VM positive area was defined as area in which at least one VM structure was detective. VM positive tumor sample was defined as a sample that contained at lease one VM positive area. Evaluation of the CD68 and COX-2 staining reaction was performed in accordance with the immunoreactive score (IRS) proposed by Remmele and Stegner: IRS = SI (staining intensity) × PP (percentage of positive cells). [31]

### In vivo human xenograft model

Four to five-week-old BALB/c-nu mice, weighing 16-18g were housed in a specific pathogen-free animal facility. Animal husbandry was performed according to the approved Sun Yat-sen University guidelines under Institutional Animal Care. Mice were divided into two groups: U87 cells without treatment and U87 cells treated with celecoxib. For intracranial transplants, 5×10^5^ U87cells in 5 ul of PBS were injected stereotaxically into the right forebrain of the mice (1 mm lateral and 1 mm anterior to bregma with 3-4 mm depth from dura). Seven days after tumor implantation, mice in treatment groups received celecoxib 100mg/kg/day po for 2 weeks.

### Survival analysis

Survival analysis of GBM patients related with COX-2 expression from TCGA dataset was performed as our previously study[32, 33]. Briefly, survival time was carried out using the Kaplan-Meier analysis and statistical significances of Progression-Free survival (PFS) were determined using the Log-Rank test. Survival analysis was performed on SPSS (version 17.0; SPSS Inc.) and the survival curve was generated by GraphPad Prism (version 5.04; GraphPad Software, Inc.). Patients who had undergone surgery, radiotherapy and chemotherapy with qualified COX-2 expression were selected, with excluding patients who had Karnofsky's score lesser than 70, and survival time lesser than 30 days, since these patients might have died for reasons other than the disease itself. A total of 458 patients who fit these criteria were included for overall and progression-free survival analysis. For stratification analysis of survival, expression levels of COX-2 were sorted by ascending orders, quartiles of 25%, 50% and 75% of the sorted COX-2 values were set as cutoffs for low expressions of COX-2. The survival time was expressed as mean ± SD.

### Statistics

Data are expressed as mean ± SD. One way ANOVA were conducted for comparisons of three or more groups about continuous variables and then followed by post hoc tests. All experiments were performed independently at least three times and in triplicate each time. All the tests were two-side, and *p* values < 0.05 were considered statistically significant. All statistical analysis was done using SPSS 17.0.
